# The contribution of Ca and Mg to the accumulation of amino acids in maize: from the response of physiological and biochemical processes

**DOI:** 10.1186/s12870-024-05287-y

**Published:** 2024-06-19

**Authors:** Zhaoquan He, Xue Shang, Xiukang Wang, Yingying Xing, Tonghui Zhang, Jianying Yun

**Affiliations:** 1 School of Life Sciences, Yan’an University, Yan’an, 716000 China; 2 Shaanxi Key Laboratory of Chinese Jujube, Yan’an University, Yan’an, 716000 China; 3 Key Laboratory of Applied Ecology of Universities in Shaanxi Province on the Loess Plateau, Yan’an University, Yan’an, 716000 China; 4 Northwest Institute of Eco-Environment and Resources, Chinese Academy of Sciences, Lanzhou, 730000 China; 5 College of Land Resource and Environment, Jiangxi Agricultural University, Nanchang, 330045 China

**Keywords:** Ca, Amino acid accumulation, Maize physiology, Mg, Defensive enzyme

## Abstract

**Background:**

The quality of maize kernels is significantly enhanced by amino acids, which are the fundamental building blocks of proteins. Meanwhile, calcium (Ca) and magnesium (Mg), as important nutrients for maize growth, are vital in regulating the metabolic pathways and enzyme activities of amino acid synthesis. Therefore, our study analyzed the response process and changes of amino acid content, endogenous hormone content, and antioxidant enzyme activity in kernels to the coupling addition of sugar alcohol-chelated Ca and Mg fertilizers with spraying on maize.

**Result:**

(1) The coupled addition of Ca and Mg fertilizers increased the Ca and Mg content, endogenous hormone components (indole-3-acetic acid, IAA; gibberellin, GA; zeatin riboside, ZR) content, antioxidant enzyme activity, and amino acid content of maize kernels. The content of Ca and Mg in kernels increased with the increasing levels of Ca and Mg fertilizers within a certain range from the filling to the wax ripening stage, and significantly positively correlated with antioxidant enzyme activities. (2) The contents of IAA, GA, and ZR continued to rise, and the activities of superoxide dismutase (SOD) and catalase (CAT) were elevated, which effectively enhanced the ability of cells to resist oxidative damage, promoted cell elongation and division, and facilitated the growth and development of maize. However, the malondialdehyde (MDA) content increased consistently, which would attack the defense system of the cell membrane plasma to some extent. (3) Leucine (LEU) exhibited the highest percentage of essential amino acid components and a gradual decline from the filling to the wax ripening stage, with the most substantial beneficial effect on essential amino acids. (4) CAT and SOD favorably governed essential amino acids, while IAA and MDA negatively regulated them. The dominant physiological driving pathway for the synthesis of essential amino acids was “IAA-CAT-LEU”, in which IAA first negatively drove CAT activity, and CAT then advantageously controlled LEU synthesis.

**Conclusion:**

These findings provide a potential approach to the physiological and biochemical metabolism of amino acid synthesis, and the nutritional quality enhancement of maize kernel.

## Introduction

Maize kernel amino acids serve as the fundamental building blocks of protein synthesis and are involved in vital physiological processes including respiration, photosynthesis, and nitrogen metabolism [1]. Consequently, they contribute significantly to the enhancement of maize quality. Calcium (Ca) and magnesium (Mg) are crucial trace elements that are indispensable for the development and growth of maize. Moreover, they perform a significant physiological function in the process of amino acid synthesis and accumulation in maize [2]. Ca and Mg can increase the rate of amino acid synthesis and regulate the ratio and metabolism of amino acids in maize, thereby increasing the amino acid content of maize kernels [3, 4]. Therefore, it is practical to investigate the physiological regulation mechanism of Ca and Mg fertilizer on the accumulation of amino acids to increase the nutritional value of maize kernels.

As a class of signaling molecules, endogenous hormones influence crop development and growth by regulating metabolic pathways, gene expression, and cell differentiation. Endogenous hormones are of significant importance in the regulation of the amino acid content of the kernel during the growth process of maize [5]. One potential consequence of endogenous hormones influencing the metabolic pathways of maize kernels is the regulation of amino acid synthesis and catabolism. In maize kernels, gibberellins (GA) can stimulate glutamate synthesis and impede glutamine catabolism, consequently leading to an elevation in glutamate content [6]. By stimulating the synthesis of methionine and lysine, indole-3-acetic acid (IAA) enhances the level of these amino acids. Furthermore, endogenous hormones might influence the level of amino acids in the kernel by regulating their transport and distribution [7]. GA can potentially increase glutamate content by facilitating the transport and distribution of glutamate to the kernel’s center [8]. On the other hand, the gene expression regulation in maize kernels by endogenous hormones can impact the synthesis and degradation of amino acids [9]. GA, for instance, can stimulate glutamate synthase gene expression, consequently enhancing glutamate synthesis. By stimulating the expression of the methionine synthase gene, IAA could boost methionine synthesis [10]. Furthermore, the activity of enzymes in the amino acid synthesis pathway can be influenced by endogenous hormones, which consequently impact the rate of amino acid synthesis. As an illustration, GA can stimulate glutamate synthetase and glutamine synthetase activity, consequently elevating the rate of glutamate and glutamine synthesis [11]. Finally, endogenous hormones can impact tissue development and cell differentiation in maize kernels, consequently influencing amino acid content and distribution. One illustration of how GA may stimulate the growth of the endosperm is by enhancing the glutamate level within the kernel [12]. IAA can increase the levels of methionine and lysine by stimulating endosperm formation. Hence, through prudent regulation of endogenous hormones, the amino acid content of maize kernels can be increased, thereby optimizing the nutritional value of maize [13]. Nevertheless, the predominant focus of ongoing research is devoted to examining the impact of exogenous hormones on amino acid synthesis regulation, whereas the exact mechanisms of endogenous hormones remain poorly comprehended [14]. Furthermore, while the majority of research has been dedicated to examining how endogenous hormones regulate gene expression in the amino acid synthesis pathway, comparatively few studies have investigated how these hormones affect the activities of enzymes involved in this process [9]. Moreover, while most of the current research concentrates on the modulation of amino acid synthesis through the action of a solitary endogenous hormone, the progression and maturation of crops frequently entail the interplay of numerous endogenous hormones [15]. Hence, it is imperative to conduct further research on the synergistic effects of multiple endogenous hormones on the regulation of amino acid synthesis in maize.

Antioxidant enzymes comprise a category of protein enzymes that safeguard cells against oxidative damage by scavenging free radicals and other detrimental oxidizing substances, thereby assisting organisms in resisting oxidative stress [16]. The nutritional level of maize is significantly influenced by the amino acid content of maize kernels, which serves as a crucial quality index. Therefore, to improve the quality of maize, it is crucial to investigate how antioxidant enzymes control the amino acid content of maize kernels [17]. It is found that antioxidant enzymes can regulate the amino acid content of maize kernels through various pathways. To begin with, antioxidant enzymes regulate redox reactions to preserve intracellular oxidative balance; these reactions, in turn, influence the metabolism and nutrient synthesis of maize kernels [18]. By scavenging free radicals and mitigating the detrimental effects of oxidative stress on maize kernels, antioxidant enzymes promote the accumulation and synthesis of amino acids and safeguard the integrity of cell membranes and proteins [19]. Additionally, antioxidant enzymes can modulate the activities of certain enzymes that are associated with amino acid metabolism. This can affect the fluxes and pathways of amino acid synthesis in maize kernels, consequently influencing the amino acid content and composition [20]. Furthermore, antioxidant enzymes can regulate the expression of genes associated with amino acid synthesis in maize kernels, thereby influencing the accumulation and synthesis of amino acids and participating in signal transduction pathways [21]. Hence, the coordination between endogenous hormones and specific regulatory pathways and physiological driving mechanisms of antioxidant enzymes, which govern the amino acid content of maize kernels, remains systematically unknown.

Ca and Mg nutrients are intimately associated with crop endogenous hormones and protective enzyme systems and play an important role in crop physiological metabolic control. It has recently been shown that maintaining a steady state of cytoplasmic-free Ca^2+^ in crops is a need for appropriate growth and development, even though Ca^2+^ concentration fluctuates dramatically in response to external stimuli like drought stress, as well as the hormones abscisic acid (ABA) and IAA [22]. Further studies showed that Ca^2+^ significantly inhibited endogenous ABA biosynthesis in maize seedlings, with the rate of inhibition proportional to Ca^2+^ content; Ca^2+^ levels displayed synergistic or antagonistic effects with IAA at different levels, which in turn changed the regulatory impact of IAA on crop growth (facilitation of IAA polar transport at Ca^2+^ > 400 mg kg^− 1^) [23, 24]. IAA, on the other hand, can enhance calcium absorption and transport. Drought increases ABA content while decreasing GA and indole propionate content in maize cells, causing an increase in cytoplasmic Ca^2+^ content by activating Ca^2+^ ion channels in the cell membrane or inducing the release of Ca^2+^ from organelles into the cytoplasm, resulting in a decrease in cell expansion pressure and adaptive stomatal closure, enhancing maize stress coordination mechanisms [25]. It was also discovered that the calmodulin (CaM) content of maize kernels was positively correlated with IAA and GA content and negatively correlated with ABA, which is a key mechanism of Ca signaling in maize tissue cells in response to external stimuli and is extremely important for investigating the physiological and metabolic characteristics of Ca regulation in maize [26]. It has also been demonstrated that Mg can influence IAA conjugation with amino acids, lowering the level of free-state IAA, and that Mg deficit causes IAA buildup in crop roots. However, the link between IAA production and metabolism, as well as Mg deficiency-induced IAA buildup, as well as the response characteristics of ABA and GA to Mg appear to be unclear and require additional investigation.

Exogenous Ca^2+^ and Mg^2+^ have also been found to significantly increase the activities of peroxidase (POD), superoxide dismutase (SOD), and catalase (CAT) in maize leaves, induce the synthesis of chlorophyll and the main osmoregulatory substance-proline (Pro), increase its content, inhibit lipoxygenase activity, and prevent active oxygen (ROS) damage to cell membrane structure [27]. However, increased POD activity can oxidize and degrade IAA, resulting in crop failure. Furthermore, adequate Mg^2+^ concentrations can minimize the accumulation of nicotinamide adenine dinucleotide phosphate (NADPH), restrict ${{\text{O}}_{2}}^{-}$formation in chloroplast active oxygen (ROS), reduce leaf malondialdehyde (MDA) level, weaken membrane lipid peroxidation, and preserve normal cytoplasmic membrane function in maize [28]. It is obvious that scientific and reasonable management of Ca and Mg fertilization, and the threshold range identification of endogenous hormone and protective enzyme activity, are critical for a favorable physiological response process of crop growth and development [29]. According to the findings, Ca and Mg fertilization can improve the stability of the biofilm system in maize by altering endogenous hormones and protective enzymes, increasing the body’s resilience to stress, and thereby controlling the physiological metabolic processes in maize [30]. However, the research on the dynamic and continuous response characteristics of endogenous hormones and protective enzymes in the regulation process of Ca and Mg fertilizers, the range of their promotional thresholds, and their synergistic relationship with Ca and Mg fertilizers are not systematic enough. In addition, the synergistic regulation process and mechanism of endogenous hormones and protective enzymes on kernel amino acids after Ca and Mg fertilizer coupling addition are still unclear.

One of the primary crops cultivated in the rainfed region of the loess plateau, which is an important agricultural region in China, is maize. Ca and Mg are in a strong alkaline mineral state and are difficult to dissolve in water in the alkaline soil of the loess plateau. As a result, maize is incapable of absorbing and utilizing them [31]. Therefore, Ca and Mg nutrients are primarily absorbed by maize from the water-soluble and substitution states. Nevertheless, on the one hand, the content of substitutional Ca and Mg in the farmland in this region is significantly lower than the limit of the threshold required for the normal growth and development of maize, coupled with the phenomenon of over-application of nitrogen, phosphorus and potassium fertilizers in recent years, which produces a significant antagonism to the absorption of substitutional Ca and Mg in maize [32]. On the other hand, the concentrated precipitation in the loess plateau during the critical reproductive stage of maize causes the majority of the trace soluble Ca^2+^ and Mg^2+^ in the soil to be lost, resulting in an even lesser final Ca and Mg absorption and utilization by the maize. Consequently, the development of physiological symptoms is impeded by a deficiency of Ca and Mg nutrients, such as leaf yellowing, spot formation, and scorching, which impede photosynthesis and the translocation accumulation of organic matter [31, 32]. Finally, the synthesis of amino acids in maize kernels is hindered. In light of this, a crucial issue of maize physiology is the resolution of the problem of how to systematically reveal the physiological driving mechanism of Ca and Mg fertilizer increase for amino acid accumulation in maize kernels. This holds practical importance in optimizing fertilizer management and maize quality control in rainfed regions, with the ultimate objective of attaining “appropriate fertilizer, superior quality, and ecological sustainability.“. Hence, the present study investigates the impact of Ca and Mg fertilizers on endogenous hormones, antioxidant enzymes, and amino acid content of maize kernels. In pursuit of the subsequent objectives: (1) to examine the synergistic relationship between endogenous hormones and protective enzymes and the regulation of Ca and Mg fertilizer; (2) to determine the systematic physiological driving mechanism of Ca and Mg-endogenous hormones-protective enzymes-amino acids for the formation of maize nutrient quality. It is anticipated that the findings of this research will address the “bottleneck” issue pertaining to the regulation of Ca and Mg fertilizers, as well as the physiological mechanism impeding maize quality improvement. Furthermore, they will furnish scientific benchmarks that can be utilized in rainfed farming regions to guide the precise management of maize fertilization and quality control.

## Methods

### Experimental scheme

#### Experimental site

It is located in the Baota district of Yan’an, Shaanxi Province, at positions 36°54′21′′N, 109°35′45′′E. It is a typical rain-fed agricultural region that experiences 540 mm of precipitation per year, concentrated primarily from July to September. The average annual temperature is 8.7℃, and there are 2421 h of sunlight and 146–179 days without frost. The loess parent material is composed of homogeneous yellow loamy soil. Additionally, it is widely exposed on the ground [33]. The fundamental physical and chemical characteristics of the soil are as follows: a pH of 8.6 in the soil layer stretching 100 cm, 6.33 g kg^− 1^ of organic matter, 0.88 g kg^− 1^ of total nitrogen, 0.64 g kg^− 1^ of total phosphorus, 19.72 g kg^− 1^ of total potassium, 16.45 mg kg^− 1^ of effective phosphorus, 145.28 mg kg^− 1^ of fast-acting potassium, and uniform soil fertility.

#### Experimental design

**Fig. 1:**
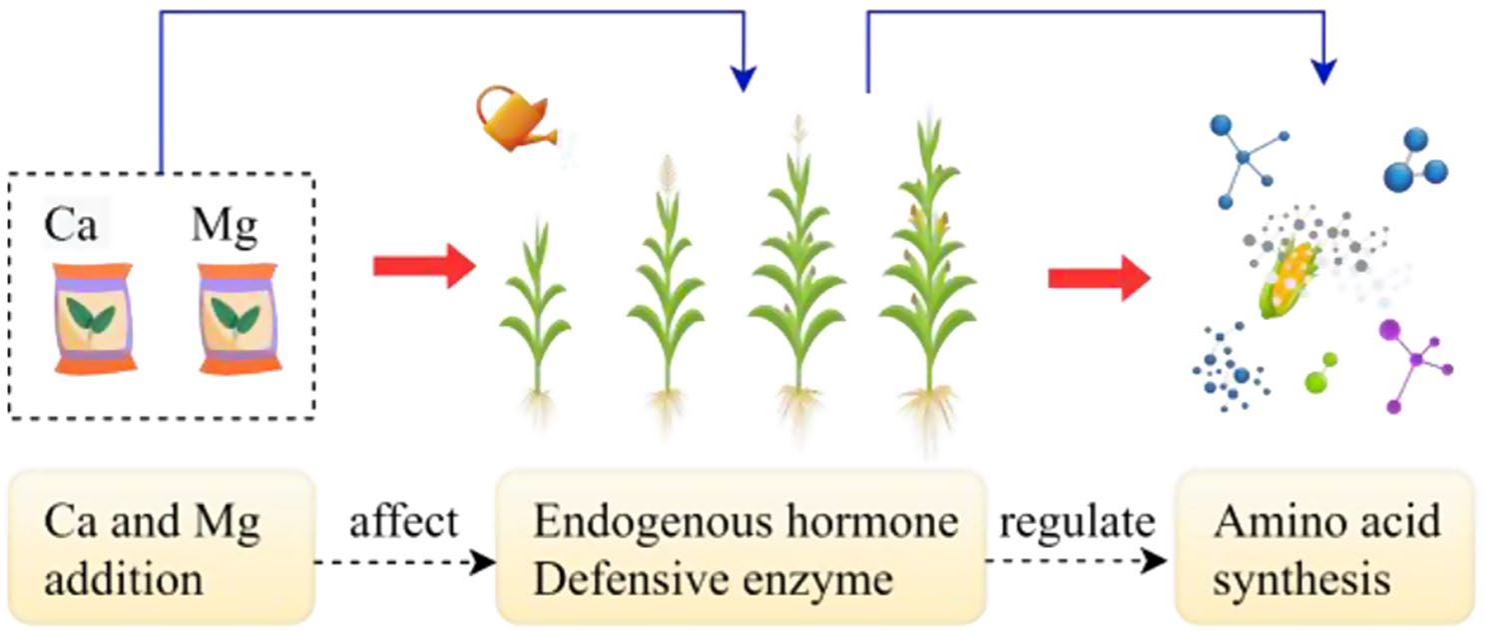
Schematic diagram of physiological processes affecting amino acid synthesis in maize kernels by coupled Ca and Mg addition. Ca and Mg represent calcium and magnesium (the same below)

The anabolism of maize kernel nutrient quality indexes can be significantly enhanced by both single Ca and Mg additions, and calcium has greater efficacy than magnesium in promoting the reproductive growth of maize, according to our previous research [32]. Therefore, the physiologically driven process and mechanism of the formation of maize kernel nutrient quality by the coupled addition of Ca and Mg are further analyzed in this study. The single-factor randomized block design was used as the experimental design. Based on the reference threshold of deficiency supplementation of maize demand for calcium (Ca) and magnesium (Mg), five treatments were designed and duplicated four times each for the entire reproductive period of maize, including T1 (treatment 1): 17.50 kg hm^− 2^ Ca + 24.50 kg hm^− 2^ Mg; T2 (treatment 2): 49.00 kg hm^− 2^ Ca + 24.50 kg hm^− 2^ Mg; T3 (treatment 3): 17.50 kg hm^− 2^ Ca + 35.00 kg hm^− 2^ Mg; T4 (treatment 4): 49.00 kg hm^− 2^ Ca + 35.00 kg hm^− 2^ Mg; CK (control treatment): no Ca and no Mg, and a total of 15 test plots were formed (Ma et al., 2012). The nutrients Ca and Mg were extracted from sugar alcohol-chelated Ca (Ca≥180 g L^− 1^, 250 g/bottle) and Mg (Mg≥120 g L^− 1^, 300 g/bottle) fertilizers, with the ionic form of the exchange and water-soluble state, which were non-toxic, easily absorbed, and beneficial to the environment. “H6281” was selected as the spring maize variety for testing due to its resistance to diseases, high yield, remarkable adaptability, and rapid dehydration of seeds. Following prior research, the following phases of maize development were supplied with Ca and Mg in the following proportions: 1:2:3:4, during the seedling-elongation, elongation-tasseling, tasseling-silking, and silking-filling stage. To ensure that maize would effectively absorb the nutrients, Ca and Mg were selected for uniform foliar spray distribution on the growth sites of above-ground organs, including leaves (including center leaves), stems, and kernels, at 16:00 h on a windless and sunny day [34]. The schematic representation is illustrated in Fig. 1.

#### Field management

The experimental design comprised 15 test plots, each measuring 6 m×7 m. Every testing region was cultivated utilizing full mulching technology, wherein the maize was directly applied onto the surface of the furrows. The thickness of the mulch, which was composed of oxidized biodegradable ecological material, was 0.008 mm. Every test plot was established at a density of 60,000 plants hm^− 2^. The furrow widths measured 30 cm, while the monopoly heights were 15 cm (with a marginal monopoly width of 10 cm). A 10 cm distance is maintained between the maize along the side of each row and the edge of the sample plot. The distance between rows of maize was 50 cm. Protection rows were positioned along the peripheries of each test plot at a distance of 5 m, and the test plots were spaced apart by 4 m, to ensure the accuracy of the results [35]. The seedlings, which had been sown on April 28, 2022, were harvested on September 25, 2022. Basal fertilizer was evenly distributed throughout all sample sites in the subsequent proportions prior to sowing: N, 130 kg hm^− 2^; P_2_O_5_, 120 kg hm^− 2^; K_2_O, 38 kg hm^− 2^) [36]. The following dates were scheduled for the topical application of sugar alcohol Ca and Mg fertilizers: May 30th, June 25th, July 10th, and July 29th. For indicator purposes, preserved maize kernels were collected and analyzed on the subsequent days following maize pollination: 8, 16, 24, 32, 40, and 48.

### Indicator measurement and methodology

#### Physiological and biochemical indicators of maize kernel

The exact dates of sample collection were as follows: September 7th, September 15th, September 21st, September 24th, and September 2nd, taking into account the impact of precipitation-filled days. For each sampling, four replicates of each treatment were selected at random. 10 g of representative kernels were selected from four maize plots each time (to ensure the dependability of the test data, non-marginal plants were specifically chosen). Almond foil, tinfoil, and clean gauze tape were utilized to affix kernel samples for each treatment, which were individually labeled and sealed. Following their expeditious freezing in a tank of liquid nitrogen, the specimens were returned to the laboratory, where they were preserved in an ultra-low temperature refrigerator set to -80 °C as a backup sample for measurement of endogenous hormones and antioxidant enzymes in maize kernel [37].

Extraction and content of endogenous hormones in kernels: Referring to indirect enzyme-linked immunosorbent assay (ELISA), each sample was repeated four times. Frozen samples were homogenized with pre-cooled 80% aqueous alcohol solution containing 1 mmol-L^− 1^BHT, immersed at 4 ℃ overnight, and then centrifuged at 10,000 rpm for 30 min. The residue was added to 1 ml of the extraction solution after the supernatant was taken, then extracted for 1 h at 4 ℃, and then centrifuged to consolidate the supernatant and record the volume. The extract was purified on a Scp2Pak C18 column to remove the pigment and other lipophilic impurities. Methanol was removed by evaporation under reduced pressure. The extracts were dissolved in TBS (50 mmol·L^− 1^Tris, 150 mmol·L^− 1^NaCl, 1.0 mmol·L^− 1^ pH 7.8 Mg Cl_2_). The absorbance was measured at 450 nm using an enzyme meter, and the contents of abscisic acid (ABA), indole-3-acetic acid (IAA), gibberellin (GA), and zeatin riboside (ZR) were calculated from the standard curve [7, 12, 18].

Antioxidant enzyme activity, malondialdehyde (MDA), and proline (Pro) content: Nine fresh spike leaves were selected for each treatment at the filling and milk ripening stages, rinsed and wiped with distilled water, wrapped in tinfoil, and stored in liquid nitrogen tanks at low temperatures for determination of antioxidant enzyme activity, MDA content and Pro content of maize leaves. 0.3 g of maize leaves were accurately weighed and were fully ground and centrifuged at 12,000 r min^− 1^ for 20 min at 4℃ after being mixed with 2.7 mL of sampling buffer and a small amount of quartz sand, and the supernatant was the crude enzyme solution, which was then taken for the determination of the enzyme activities, and the activities of peroxidase (POD), catalase (CAT), superoxide dismutase (SOD), and ascorbic acid peroxidase (APX) was measured by the colorimetric method of guaiacol, UV-absorbent method, NBT photo-reduced method, and the micro method, respectively; Pro content and MDA content were determined by sulfosalicylic acid method and thiobarbituric acid method, respectively [22, 26, 38].

#### Ca and Mg of maize kernel

Gathering 5 g of disease-free, fresh kernels from four non-marginal maize plots. Before being dried to a constant weight at 100 °C, the grains underwent natural desiccation after a thorough mixing process. For Ca and Mg analysis, retaining maize kernels that have been ground and crushed with an automatic ball mill and a 60-mesh sieve [39].

A single determination was conducted using 1 g of maize kernel sample powder (with an accuracy of 0.0001 g). Following a series of procedures including hydrochloric acid digestion, cooling, filtration, and HNO_3_-HClO_4_ elimination, the sample powder was transferred to a 100mL volumetric flask and thoroughly mixed. Following the addition of 0.50 mL of the internal standard solution (100 µg L^− 1^) to 10 mL of the sample solution via pipette, the Ca and Mg concentrations in the kernels were determined utilizing the filtrate in conjunction with an inductively coupled plasma emission spectrometer (ICP, AES-iCAP6300, Thermo Fisher Scientific, MA, USA) [40].

#### Essential amino acids and their components of maize kernel

A quantity of 10 g of fresh, healthy kernels was obtained from four maize plants in each test plot at each time, excluding marginal plants. Following their combination and sealing in a preserving apparatus (a refrigerated icebox), these kernels were expeditiously returned to the laboratory. The samples were dried through a natural occurrence. After reducing the moisture content of the samples to 14% ± 1%, the kernels were dried at 60 °C for an extended period until their weight remained constant. The kernels were subjected to a series of procedures, including crushing, sieving through a 100-micron sieve, and desiccator storage, to determine the essential amino acid and component contents, including essential amino acid (AMA), valine (VAL), methionine (MET), isoleucine (ISO), leucine (LED), phenylalanine (PHE), lysine (LYS), threonine (THR) [41]. For each indicator, each of the aforementioned measures was examined four times.

The amino acid content of maize kernel: dry and grind maize kernel into powder, weigh 0.5 g of sample, add 0.001 M HCL 20% ethanol solution 3 ml, ultrasonic in a low-temperature water bath for 30 min, centrifuged at 12,000 g at 4℃ for 5 min, take the supernatant, add 1 ml of precipitate extract to re-extract two times. Finally, the three supernatants were combined to 10 ml. 1 ml of liquid was aspirated, passed through 0.22 μm aqueous filter membrane, and put into the refrigerator at -20 ℃ for online testing. The amino acid content of the sample was determined by HPLC-MS/MS (Agilent 1260 liquid chromatography coupled with AB4000 mass spectrometer) [20].

### Analysis software and methods

The experimental data was statistically analyzed using SPSS 19.0, Origin Pro 2021, and R 3.5.2. One-way analysis of variance (ANOVA) followed by the least significant difference (LSD) test was used to compare the differences of all treatments in kernel Ca, Mg, endogenous hormones, antioxidant enzyme, essential amino acid, and its components, respectively, after maize pollination. network heatmap with a significance level of *P* < 0.05 was used to determine the degree of correlation between Ca, Mg, endogenous hormones, and antioxidant enzymes for each treatment. The variation characteristics of essential amino acids and their components in each treatment were analyzed by box plot. The proportion of each essential amino acid component was analyzed by cumulative histogram, and the degree of their influence on the synthesis of essential amino acids was determined by principal component analysis. The redundancy analysis was used to determine the degree of influence of Ca, Mg, endogenous hormones, and antioxidant enzymes on essential amino acids and their components. The structural equation model was used to reveal the dominant driving pathway of essential amino acid synthesis.

## Results

### Changes of Ca and Mg elements, physiological and biochemical indexes in maize kernels, and their response relationships

**Fig. 2:**
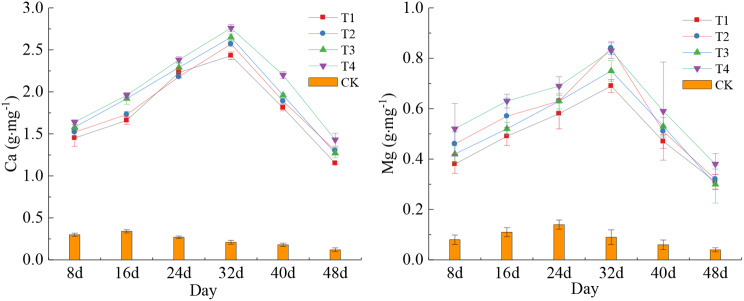
Characteristics of changes in Ca and Mg contents of maize kernels in different treatments. Data represent mean ± standard deviation (*n* = 4). Ca and Mg represent calcium and magnesium (the same below)

As shown in Fig. 2, the Ca and Mg contents of maize kernels in treatments T1, T2, T3, and T4 exhibited a trend of initial increase followed by subsequent decrease starting from the filling stage. The maximum value of Ca and Mg was observed on the 32nd day after maize pollination, corresponding to the waxing and ripening stage. At this time, the Ca contents of the above treatments were as follows: 2.43 g · mg^− 1^, 2.57 g · mg^− 1^, 2.65 g · mg^− 1^, and 2.76 g · mg^− 1^. The Mg contents of them were as follows: 0.69 g · mg^− 1^, 0.84 g · mg^− 1^, 0.85 g · mg^− 1^, and 0.83 g · mg^− 1^. While T4 contained the highest contents of Ca and Mg in its kernel, T1 had the lowest contents of both Ca and Mg, the differences between the two were not statistically significant. The Ca and Mg contents in the kernels of CK exhibited a persistent decline. Specifically, both Ca and Mg values peaked at 0.12 g · mg^− 1^ and 0.04 g · mg^− 1^, respectively, on the 48th day after maize pollination. Notably, these values were significantly lower than those of T1-T4 (*P* < 0.001). This finding suggested that the simultaneous application of varying concentrations of Ca and Mg fertilizers resulted in a substantial increase in the elemental contents of Ca and Mg in maize kernels. The maximum value of Ca and Mg was observed during the waxy ripe stage of maize, after which there was a gradual decline in content until the full-ripe stage. The content of Ca and Mg in kernels was directly proportional to the amount of Ca and Mg fertilizer added, within a certain range.

**Fig. 3:**
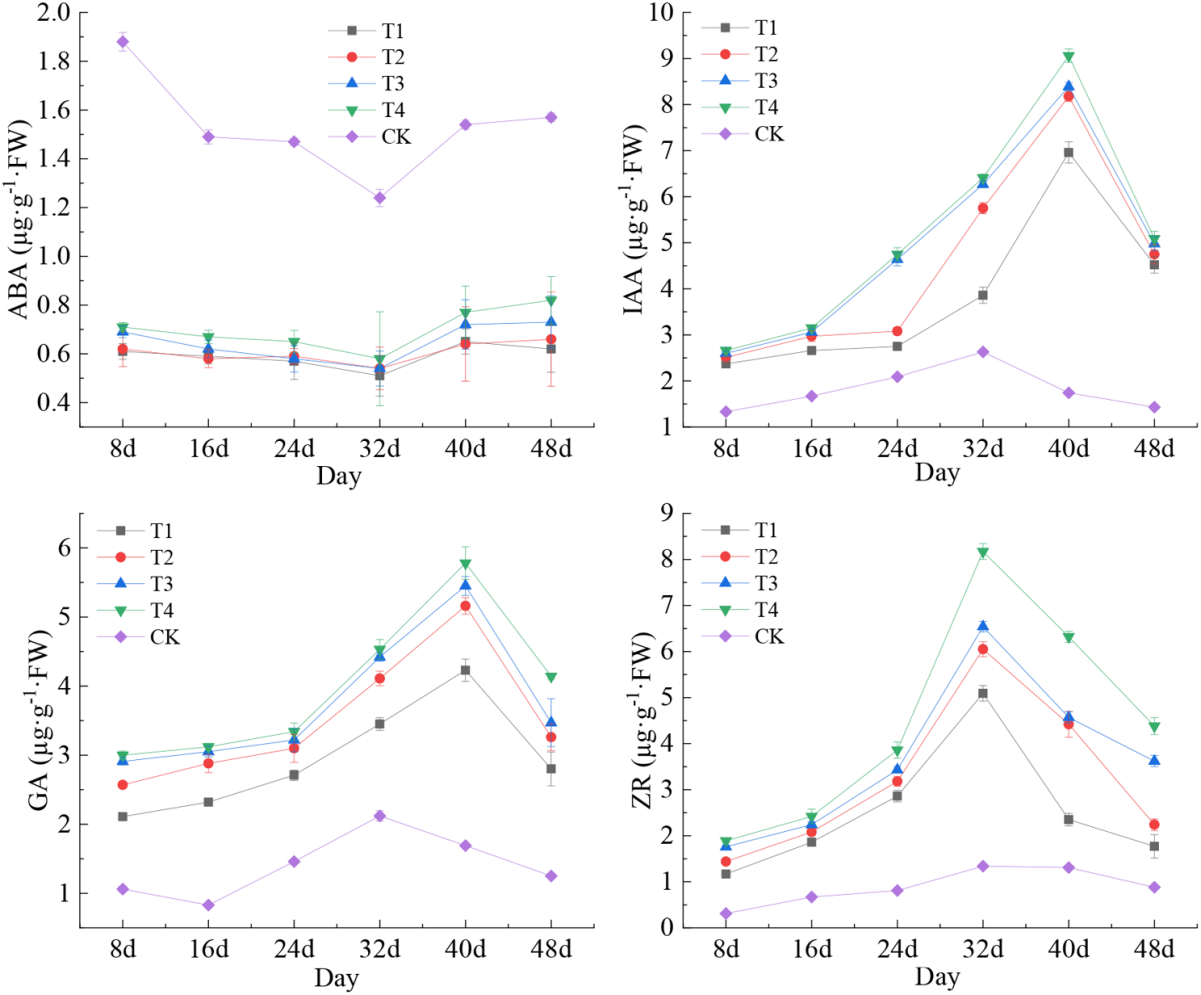
Characteristics of changes in endogenous hormone contents of maize kernels in different treatments. Data represent mean ± standard deviation (*n* = 4). ABA, IAA, GA, and ZR represent abscisic acid, indole-3-acetic acid, gibberellin, and zeatin riboside, respectively (the same below)

In general, the variations in ABA contents in kernels of T1-T4 were minimal from the filling to maturity stages of maize. However, the contents of IAA, GA, and ZR in T1-T4 exhibited an apparent trend of initial increase followed by subsequent decrease, with significant fluctuations commencing 24 days after maize pollination (Fig. 3). The content of ABA in kernels of CK exhibited a distinct trend of decline followed by an increase, whereas the variability of the other endogenous hormones of CK was minimal. The minimum value of ABA was observed in T1-T4 on the 32nd day (the waxing ripe stage) after pollination. Notably, this difference from the value of CK was statistically significant (*P* < 0.001). The maximum IAA contents of T1-T4 were observed on the 40th day after maize pollination. The sequential values of these values were as follows: 6.96 µg·g^− 1^·FW, 8.18 µg·g^− 1^·FW, 8.39 µg·g^− 1^·FW, and 9.06 µg·g^− 1^·FW. Notably, the difference between these values and those of CK (1.74 µg·g^− 1^·FW) was substantial (*P* < 0.001). The maximum values of GA for T1-T4 were as follows: 4.23 µg·g^− 1^·FW, 5.16 µg·g^− 1^·FW, 5.45 µg·g^− 1^·FW, and 5.78 µg·g^− 1^·FW, respectively, on the 40th day following maize pollination. The difference between these values and the GA content of CK (1.69 µg·g^− 1^·FW) was found to be statistically significant (*P* < 0.001). The maximum ZR content of T1-T4 was observed on the 32nd day following maize pollination; this difference was significantly larger (*P* < 0.001) in comparison to the ZR content of CK. Generally, the magnitudes of the differences between treatments regarding endogenous hormone contents of maize kernels subsequent to the combined application of Ca and Mg fertilizers were as follows: T4 > T3 > T2 > T1. However, these differences did not reach statistical significance. This finding suggested that the simultaneous application of Ca and Mg fertilizers led to a substantial increase in the content of endogenous hormones in the kernel. Specifically, the levels of IAA, GA, and ZR exhibited a sustained upward trend from the filling stage to the wax ripening stage, whereas ABA displayed a less volatile fluctuation, which was advantageous for the development and growth of maize.

**Fig. 4:**
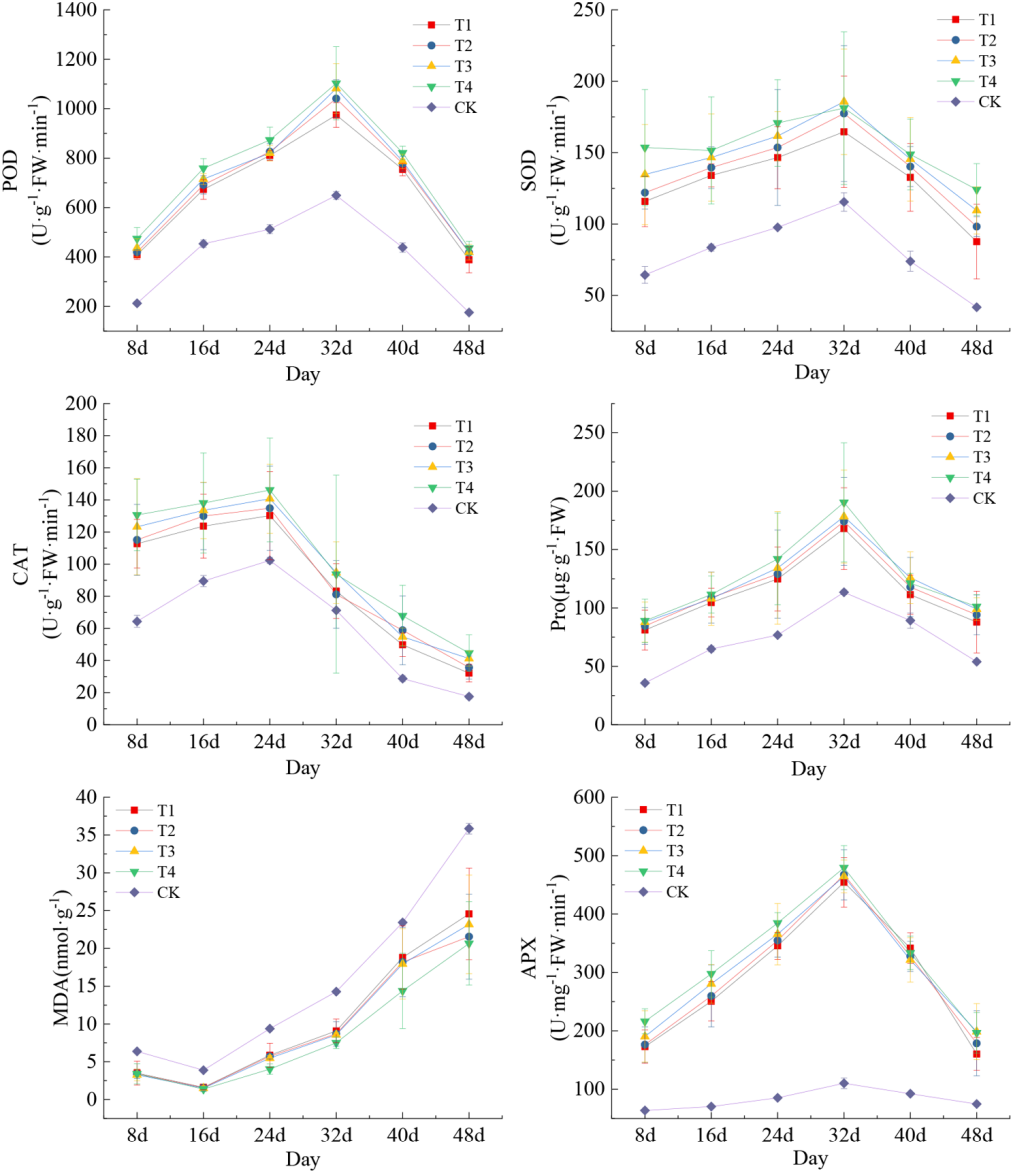
Characteristics of changes in protective enzyme activities of maize kernels in different treatments. Data represent mean ± standard deviation (*n* = 4). POD, SOD, CAT, APX, Pro, and MDA represent peroxidase, superoxide dismutase, catalase, ascorbic acid peroxidase, proline, and malondialdehyde, respectively (the same below)

As shown in Fig. 4, the POD, SOD, Pro, APX contents and enzyme activities of the kernels of treatments T1, T2, T3, T4, and CK increased continuously from the maize filling stage, reached the maximum value on the 32nd day after pollination (waxing stage), and then showed a continuous decreasing trend. The peak activity of kernel CAT was observed in all treatments on the 24th day subsequent to maize pollination, after which it declined substantially. The maximum value of MDA content in maize kernels was reached on the 48th day following maize pollination, with a consistent increase observed in all treatments starting from the filling stage. In general, the content and activities of POD, SOD, CAT, Pro, and APX in T1, T2, T3, and T4 were found to be significantly greater than those in CK (*P* < 0.001), whereas their MDA contents were marginally lower (*P* < 0.01). However, there were no significant differences observed in the levels of antioxidant enzymes among treatments T1, T2, T3, and T4. This result suggested that the simultaneous supplementation of varying contents of Ca and Mg led to a substantial enhancement in the activities of antioxidant enzymes in maize kernels. Notably, these enzyme activities peaked during the wax ripening stage of maize, thereby substantially bolstering the cells’ resistance to oxidative damage. However, the sustained increase in MDA observed in the time series after the filling stage would pose a partial challenge to the cellular membranoplasmic defense system.

**Fig. 5:**
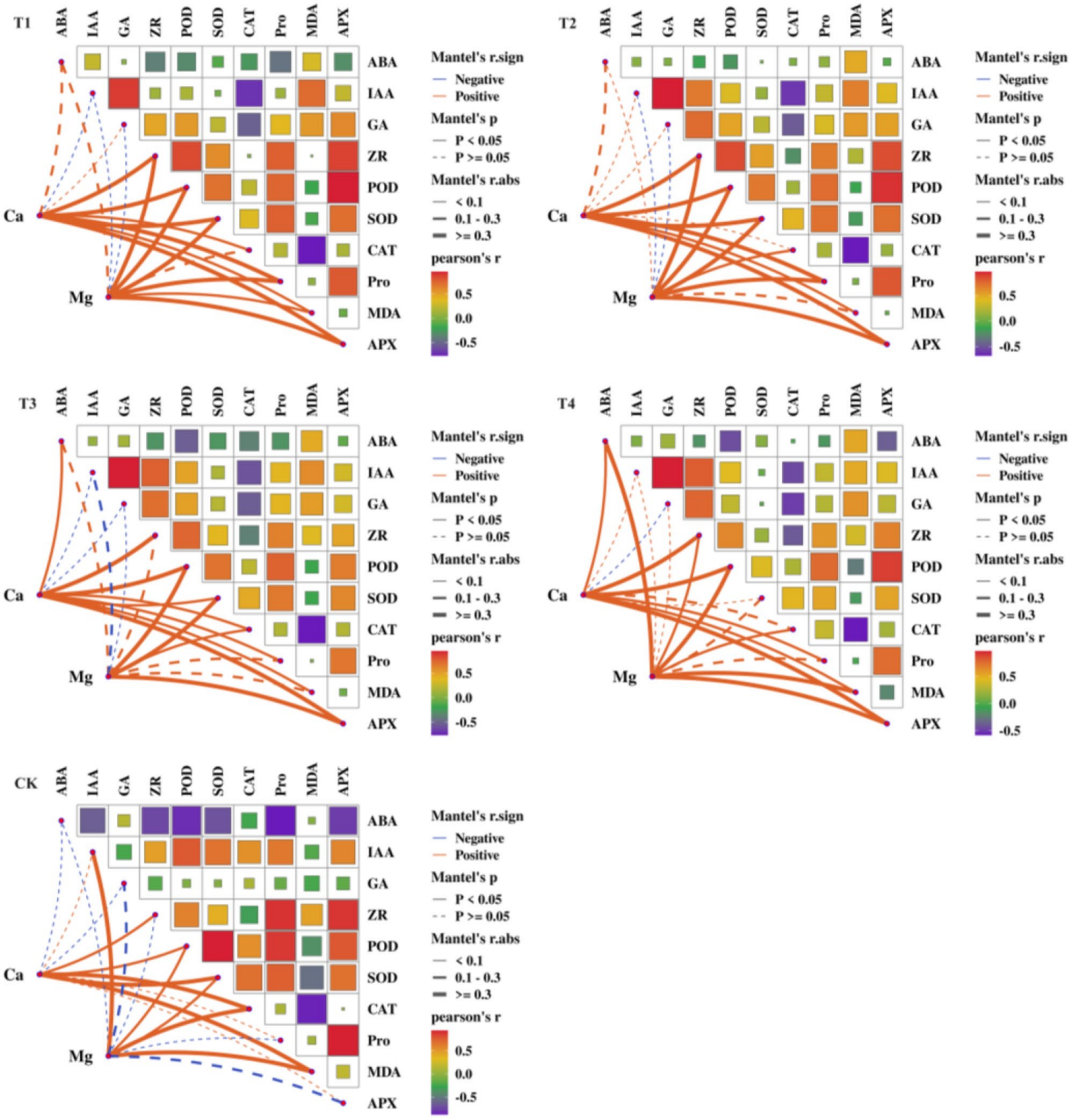
Correlation heat map of Ca and Mg content of maize kernels with endogenous hormones and antioxidant enzymes. Data represent mean ± standard deviation (*n* = 24)

The correlation heat map revealed that Ca and Mg exhibited highly significant positive correlations with ZR, POD, SOD, Pro, and APX in T1 and T2, while ZR demonstrated significant positive correlations with POD, SOD (Fig. 5), Pro, and APX. Highly significant positive correlations were observed between Ca and Mg and POD, SOD, and APX in T3. Furthermore, significant positive correlations were identified among the aforementioned antioxidant enzymes. Highly significant positive correlations were observed between Ca and Mg and ABA, ZR, POD, MDA, and APX in T4. Conversely, ABA exhibited significant negative correlations with POD, MDA, and APX. MDA exhibited significant negative correlations with SOD and CAT, whereas Ca and Mg demonstrated highly significant positive correlations with SOD, CAT, and MDA in CK. This finding suggested that the coupled addition of varying levels of Ca and Mg fertilizer significantly increased the activities of antioxidant enzymes in maize kernels. Furthermore, the correlation between Ca, Mg, and antioxidant enzyme activities was significantly stronger than that observed with endogenous hormones.

### Changes of essential amino acids and their components in maize kernels

**Fig. 6:**
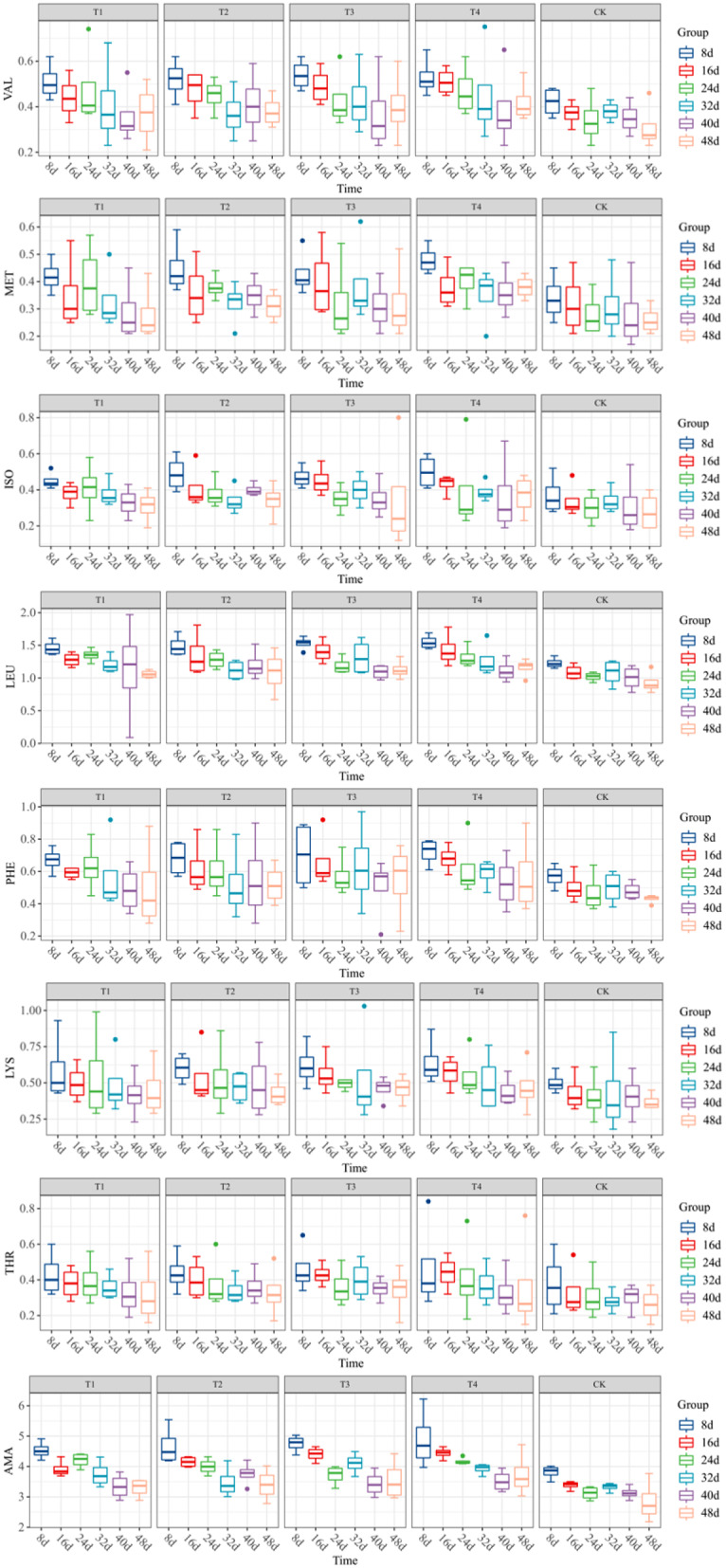
Changing characteristics of the content of essential amino acid components in maize kernels of different treatments. Data represent mean ± standard deviation (*n* = 4). VAL, MET, ISO, LED, PHE, LYS, THR, and AMA represent valine, methionine, isoleucine, leucine, phenylalanine, lysine, threonine, and essential amino acid, respectively (the same below)

As indicated by the change characteristics of each essential amino acid component, the AMA and its components in T1-T4 exhibited a progressive decline in the 48 days after maize pollination after the coupled addition of Ca and Mg. This trend was consistent with that of CK. This suggested that the amino acid content of the kernel decreases steadily from the filling stage to the maturity stage of maize. T1 had a significantly lower MET content than T2-T4 between the 32nd and 48th day following maize pollination, and its MET content was notably distinct from that of T4. The VAL content of T3 and T4 decreased substantially over the time series. The LEU exhibited comparable variability across all treatments; the mean LEU values for T1-T4 were 1.25, 1.26, 1.28, and 1.31, respectively, which were significantly greater than the value for CK at 1.06. PHE induced the most substantial reduction in CK and the least reduction in T1. The reductions in LYS and THR were similar across all treatments, indicating a relatively smooth decline (Fig. 6). In general, the coupled addition of Ca and Mg did not yield a statistically significant difference between AMA and its components in T1-T4. However, the difference was significantly greater than that in CK. It suggested that the simultaneous addition of varying contents of Ca and Mg would marginally increase the essential amino acid content of maize kernels.

**Fig. 7:**
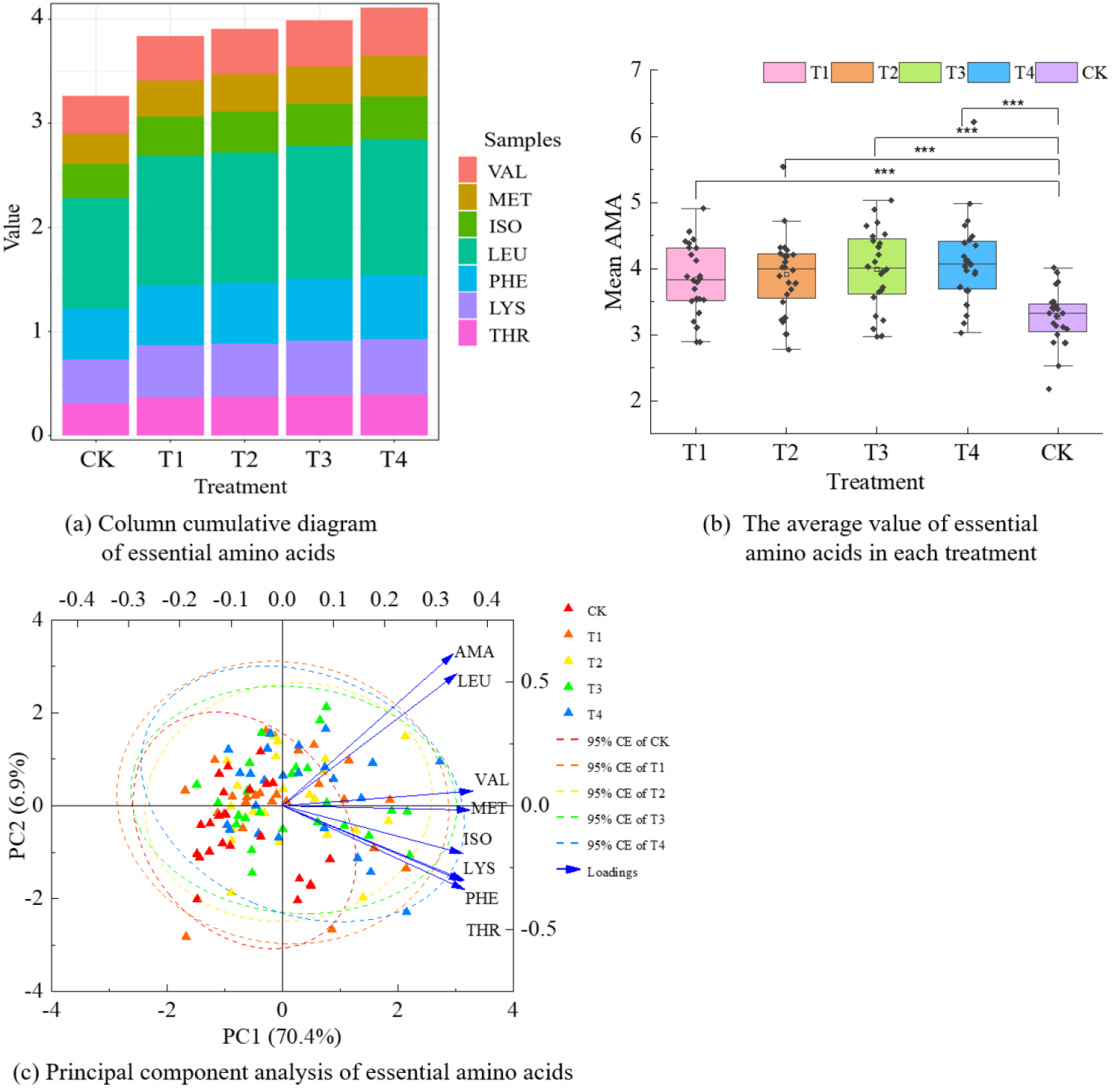
Column cumulative plots (**a**), average value characteristic (**b**), and principal component analysis (**c**) of the contents of essential amino acid components of maize kernels from different treatments (*n* = 24). In (**a**), the value represents the mean value of each essential amino acid component; in (**b**), data represent mean ± standard deviation (*n* = 24), *** represents *P* < 0.001; in (**c**), *n* = 24 in each treatment, CE represents a confidence ellipse.

The contents of essential amino acid components in maize kernels for each treatment were as follows, as shown in Fig. 7(a): LEU > PHE > LYS > VAL > ISO > THR > MET, and LEU was significantly higher in content than the other essential amino acid components. The magnitudes of the mean values of essential amino acids for each treatment were as follows: T4 > T3 > T2 > T1 > CK, which were 3.84, 3.91, 3.99, 4.11, and 3.27, respectively, as shown in Fig. 7(b). Notably, the mean values for essential amino acids in T1-T4 were significantly higher than those in CK (*P* < 0.001). It was determined that LEU, VAL, and MET significantly and positively regulated the essential amino acid of maize kernel, based on the results of the principal component analysis. Furthermore, the sequence of these components’ effects on the essential amino acid was as follows: LEU > VAL > MET (Fig. 7(c)). Therefore, it was apparent that LEU constituted the most substantial proportion of the essential amino acid components in the kernel and exerted the most pronounced positive influence on the essential amino acids.

### The physiological mechanisms of the synthesis of essential amino acids in maize kernels

**Fig. 8:**
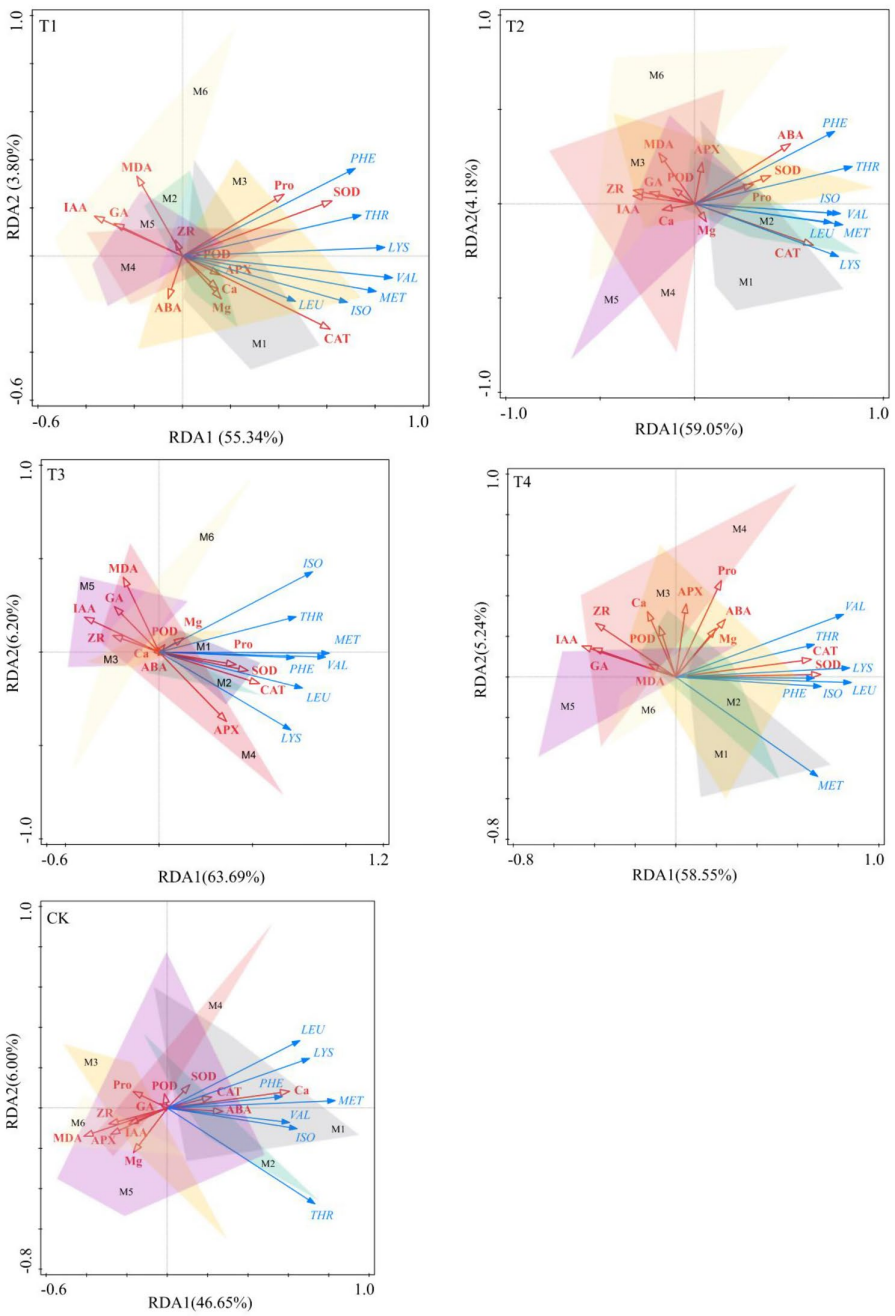
Redundancy analysis of physiological driving indexes of essential amino acid components of maize kernels in response to the coupled addition of Ca and Mg in different treatments. Data represent mean ± standard deviation (*n* = 24). M1-M5 represents the six times when the indicators are measured after the pollination of maize

The redundancy analysis revealed that in T1, CAT, SOD, and Pro regulated essential amino acids significantly and positively, whereas MDA, IAA, and GA substantially and negatively regulated essential amino acids (Fig. 8). IAA, ZR, and MDA exhibited significant negative regulatory effects on essential amino acids in T2, whereas CAT, SOD, and ABA demonstrated significant positive regulatory effects. Positive regulatory effects of CAT, SOD, and APX on essential amino acids were observed in T3, whereas significant negative regulatory effects were attributed to MDA, IAA, and GA on essential amino acids. Essential amino acids were positively regulated in T4 by CAT, SOD, and Pro, while they were significantly negatively regulated by IAA, GA, and ZR. Ca positively regulated essential amino acids in CK, whereas Mg, ZR, MDA, and APX negatively regulated them significantly. In brief, CAT and SOD exhibited positive regulation of critical amino acids in maize kernels after coupled addition (T1-T4) of varying Ca and Mg contents. Conversely, IAA and MDA demonstrated negative regulation of essential amino acids in kernels.

**Fig. 9:**
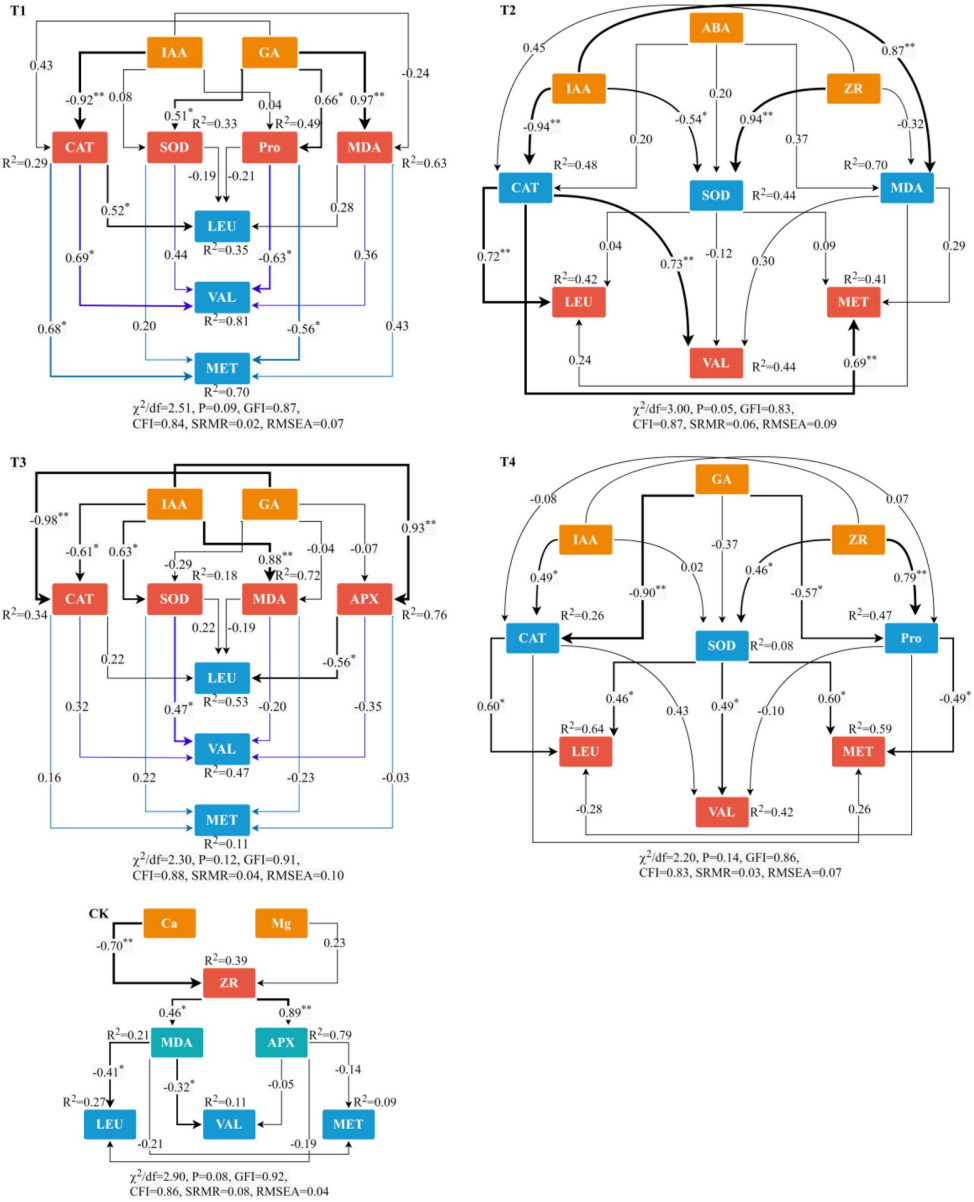
Structural equation modeling of the physiological driving pathways of essential amino acids in maize kernels in response to the coupled addition of Ca and Mg in different treatments (*n* = 24). ^*^ represents *P* < 0.05, ^**^ represents *P* < 0.01; χ^2^ represents the chi-square; df represents the degree of freedom; GFI represents the goodness of fit index; CFI represents the comparative fit index; RMSEA represents the root mean square error of approximation

The results of constructing structural equation models utilizing the primary physiological indicators and amino acid types were shown in Fig. 9. In both T1 and T2, the primary physiological driving pathways of essential amino acids were identical: IAA first inhibited CAT activity, and CAT subsequently increased the synthesis and accumulation of LEU, VAL, and MET. In contrast to T1 and T2, the amino acid synthesis driving pathways in T3 diverged from those in T1 and T2. In T3, the synthesis and accumulation of LEU were initially regulated negatively by APX, after its positive regulation of APX activity by IAA. The two primary physiological driving pathways of amino acids in T4 were as follows: GA negatively regulated CAT activity, and CAT subsequently positively regulated LEU synthesis; ZR initially positively regulated Pro content, and subsequently Pro negatively regulated MET synthesis and accumulation. Ca exhibited a substantial adverse effect on the content of ZR in CK, whereas ZR positively regulated the content of MDA, which subsequently negatively regulated LEU synthesis. In summary, IAA was the primary endogenous hormone that influenced the synthesis of amino acids in maize kernels, while CAT was the primary antioxidant enzyme following the simultaneous addition of varying Ca and Mg contents.

## Discussion

### Characteristics of endogenous hormones and protective enzymes in maize in response to synergistic application of Ca and Mg fertilizers

Ca, being an essential element for plant development and growth, facilitates the elongation and division of plant cells, thereby enhancing the capacity of plants to absorb nutrients and engage in photosynthesis [32]. Mg is a critical constituent within the molecular framework of chlorophyll, facilitating its synthesis and subsequently enhancing photosynthesis. This finally enhances the antioxidant capacity and photosynthetic efficiency of plants, and the growth of maize and enzyme activities are both positively impacted by it [23].

Endogenous hormones, such as IAA, which promotes kernel differentiation and development, and GA, which stimulates expansion and growth, are crucial in regulating the growth and development of maize kernels [42]. The present study observed that the coupling application of Ca and Mg fertilizers resulted in a transient increase and decrease in the contents of IAA, GA, and ZR in maize kernels from the filling stage to the maturity stage. The metabolism and distribution of plant growth factors may be responsible for this trend. The acceleration of maize kernel growth during the filling stage necessitates the application of substantial quantities of IAA, GA, and ZR to stimulate cell division and elongation [43]. Consequently, they undergo greater synthesis and accumulation, which initiates a pattern of escalating their contents. Nevertheless, with the advance of maturity, there is a gradual deceleration in the growth rate of maize kernels, accompanied by a gradual balancing of hormone metabolism in the maize and a corresponding reduction in the demand for IAA, GA, and ZR [40]. In contrast, maize kernels may accumulate an excessive amount of IAA, GA, and ZR due to an overabundance of Ca and Mg fertilization. This results in negative feedback regulation and a gradual reduction in the contents of these substances [44]. Lastly, the transport and absorption of IAA, GA, and ZR by maize may be impeded by changes in soil pH caused by excessive utilization of Ca and Mg fertilizers [45]. Furthermore, the concurrent application of Ca and Mg fertilizers could potentially induce alterations in the functioning of soil microorganisms, thereby influencing the processes of endogenous hormone synthesis and catabolism in maize. Consequently, this could impact the contents of IAA, GA, and ZR in maize kernels [46].

Antioxidant enzymes are crucial to the growth of maize kernels. Oxidative stress increases gradually during the development of the maize, resulting in the accumulation of oxidative damage within the cell [38]. The utilization of antioxidant enzymes in maize can effectively mitigate oxidative stress and prevent the development of oxidative damage. Following the coupling application of Ca and Mg fertilizers to maize in this study, the CAT and SOD enzyme activities of the kernels exhibited a temporal pattern characterized by an initial increase followed by a subsequent decrease from the filling to maturity stage. The reason for this is that Ca and Mg fertilizers regulate the synthesis and metabolism of endogenous hormones, thereby influencing the enhancement of antioxidant enzyme activities [47]. As Important stress tolerance and growth regulators, endogenous hormones can influence the antioxidant capacity, nutrient distribution, and growth rhythm of maize. Consequently, the characteristics of endogenous hormones and protective enzymes of the kernel are identical in response to Ca and Mg fertilization [48].

### Response characteristics of essential amino acids and their components in maize kernels to the synergistic increase of Ca and Mg fertilizers

Essential amino acids are non-bioavailable to the body and must be acquired from food sources. As a result, the essential amino acid content of maize kernels is crucial for human nutrition [49]. The addition of Ca and Mg fertilizers to a greater extent resulted in a substantial increase of the essential amino acid and component content in maize kernels. This is partially attributable to the fact that Ca and Mg are involved in material metabolism, respiration, and photosynthesis, all of which are essential for the regulation of maize growth, thereby facilitating maize development [50]. On the other hand, this phenomenon is closely related to the impact of Ca and Mg on the nitrogen metabolism of maize. Nitrogen is essential for the amino acid composition, which is responsible for the fundamental building blocks of protein synthesis in plants [20, 51]. By increasing the application of Ca and Mg fertilizers, the nitrogen efficiency in the soil can be regulated, leading to improved nitrogen utilization and absorption by maize. This, in turn, stimulates the synthesis of amino acids in maize, ultimately resulting in a higher content of essential amino acids and their components in maize kernels [31].

A gradual decline in the content of essential amino acids is observed in maize from the filling to the maturity stage. This is a result of the gradual decline in endogenous hormone contents from filling to maturity, which inhibits the kernel’s development [52]. Simultaneously, the decline in endogenous hormone levels impacts the process of essential amino acid synthesis and accumulation, culminating in further depletion of essential amino acid content [53]. Furthermore, the concurrent application of Ca and Mg fertilizers has the potential to enhance the activity and synthesis of antioxidant enzymes in maize, thereby enhancing the overall antioxidant capacity of plants [29]. Nevertheless, from filling to maturity, the activity of antioxidant enzymes in maize decreases gradually, increasing the risk of oxidative cell damage. This damage disrupts the synthesis and accumulation of essential amino acids [19, 54].

LEU, being the largest proportion among the essential amino acid components of the kernel, exerted the most significant influence on the essential amino acids [4]. It is found that LEU exhibits a significant impact on the growth of maize and the synthesis of protein. The application of Ca and Mg fertilizer to maize results in an increase in the activity of nitrogen metabolism, which thereby facilitates the synthesis and accumulation of leucine [44]. Meanwhile, Mg can stimulate the activity of enzymes involved in nitrogen metabolism within the plant, thereby increasing the rate of leucine synthesis. Therefore, the highest content of LEU in maize kernels can be attributed to the nitrogen metabolism enhancement induced in maize by Ca and Mg fertilizers [46].

### Physiological regulation mechanism of essential amino acid synthesis in maize kernel by the synergistic addition of Ca and Mg fertilizer

After the synergistic addition of Ca and Mg, IAA is the principal endogenous hormone influencing the synthesis of amino acids in maize kernels, while CAT is the principal antioxidant enzyme [55]. The regulatory pathway is “Ca, Mg-IAA-CAT-LEU,” with IAA negatively regulating the activity of CAT initially, and CAT regulating the synthesis and accumulation of LEU in a positive manner [56]. This study addresses the physiological mechanisms by which IAA and CAT regulate the levels of essential amino acids in maize kernels. Ca and Mg elements stimulate nitrogen metabolism in maize, which increases the synthesis and accumulation of IAA in maize, from the standpoint of IAA regulation [57]. In the interim, Ca controls the activities of enzymes within the plant, thereby influencing the processes of IAA synthesis and degradation. IAA subsequently regulates the activity of crucial enzymes in the amino acid synthesis pathway, thereby influencing amino acid synthesis [58]. For instance, IAA can stimulate the synthesis of crucial precursors for protein synthesis, including phenylalanine and tryptophan, in maize kernels. Furthermore, by directly regulating the expression of related genes and the activity of other key enzymes in the amino acid synthesis pathway, such as glutamate synthase and alanine synthase, IAA can influence the synthesis of additional amino acids in maize kernels [59]. By regulating the metabolism of hydroperoxides, CAT influences the amino acid synthesis process in maize kernels from the standpoint of CAT regulation. CAT initially controls the synthesis of amino acids by degrading hydrogen peroxide [14]. Hydrogen peroxide, which accumulates in plant cells and causes cellular oxidative damage, is a prevalent oxidative stressor. Hydrogen peroxide has a significant impact on the activity of amino acid synthase. Therefore, an excess of hydrogen peroxide will inhibit the activity of amino acid synthase, thereby interfering with amino acid synthesis [27]. Hydrogen peroxide can be decomposed by CAT, an essential antioxidant enzyme, into oxygen and water, thereby decreasing its concentration and mitigating the inhibitory impact of oxidative stress on amino acid synthesis. Additionally, CAT can modulate amino acid synthesis through its impact on the gene expression of amino acid synthase [31]. An increase in the level of gene expression of amino acid synthase was observed in response to CAT overexpression, which consequently stimulated amino acid synthesis. On the contrary, the absence of CAT results in the suppression of amino acid synthase gene expression, thereby influencing the amino acid synthesis process [9].

The aforementioned mechanistic explanation unequivocally validates the indispensable role that IAA and CAT play in the regulation mechanism of amino acids in maize kernels in response to Ca and Mg fertilizer [21]. Undoubtedly, the modulation of CAT enzyme activity by IAA influences the regulation of kernel amino acids, and the mechanism by which IAA regulates CAT enzyme activity may encompass numerous signal transduction pathways [60]. IAA can regulate the expression and activity of the CAT in plant cells by activating a series of signaling pathways [15]. In maize, these signaling pathways may involve the activation and response to exogenous environmental factors, as well as the synthesis of endogenous hormones. Hence, the complementary and indispensable role of the regulatory effects of IAA and CAT on kernel amino acids is established.

## Conclusion

Our study investigated the physiological and biochemical driving processes and mechanisms of the coupled addition of Ca and Mg fertilizers on amino acid synthesis in maize, and the main conclusions were as follows:

The coupled addition of Ca and Mg fertilizers significantly increased the content of Ca, Mg, endogenous hormone components (IAA, GA, and ZR), and antioxidant enzyme activities in kernels. The contents of IAA, GA, and ZR continued to rise and the activities of SOD and CAT were elevated from the filling to the wax ripening stage. However, the MDA content showed a sustained increase, which would attack the defense system of the cell membrane plasma to some extent.

The content of essential amino acids and their components in maize kernels was to a certain extent elevated by the simultaneous addition of Ca and Mg fertilizers, but their contents showed a gradual decrease in the time series from the filling to the maturity stage. Among them, LEU had the largest percentage and the most significant degree of positive effect on essential amino acids.

CAT and SOD exhibited substantial positive regulation of kernel essential amino acids after the simultaneous addition of varying Ca and Mg levels, whereas IAA and MDA exhibited substantial unfavorable modulation. The dominant physiological driving pathway for the synthesis of essential amino acids was “IAA-CAT-LEU”, in which IAA first negatively drove CAT activity, and CAT further positively drove the accumulation of LEU.

## Data Availability

Data will be made available on request.

## Abbreviations

Ca: Calcium
Mg: Magnesium
ABA: Abscisic acid
IAA: Indole-3-acetic acid
GA: Gibberellin
ZR: Zeatin riboside
POD: Peroxidase
SOD: Superoxide dismutase
CAT: Catalase
APX: Ascorbic acid peroxidase
Pro: Proline
MDA: Malondialdehyde
ROS: Active oxygen
VAL: Valine
MET: Methionine
ISO: Isoleucine
LEU: Leucine
PHE: Phenylalanine
LYS: Lysine
THR: Threonine
AMA: Essential amino acid
NADPH: Nicotinamide adenine dinucleotide phosphate
CaM: Calmodulin
